# Atherosclerosis Burden in Patients with Acute Chest Pain: Obesity Paradox

**DOI:** 10.1155/2014/634717

**Published:** 2014-01-12

**Authors:** Kongkiat Chaikriangkrai, Mahwash Kassi, Sayf Khaleel bala, Faisal Nabi, Su Min Chang

**Affiliations:** ^1^Department of Medicine, The Methodist Hospital, Houston, TX 77030, USA; ^2^Methodist DeBakey Heart & Vascular Center, The Methodist Hospital, Houston, TX 77030, USA

## Abstract

Obesity paradox has been described in various populations of coronary artery disease, mainly asymptomatic subjects. However, relationship between obesity and coronary artery calcification detected by cardiac CT in symptomatic patients has rarely been demonstrated. This study seeks to investigate whether the paradoxical relationship between obesity and coronary artery calcification exists in patients with acute chest pain. A final cohort of 1030 chest pain patients presenting at our emergency department who underwent coronary evaluation by multidetector cardiac CT were examined. With absent-to-mild coronary calcification (CAC score < 100) as a referent, multivariable analysis showed that presence of obesity (OR 0.564; 95% CI 0.395, 0.806; *P* 0.002), body mass index (OR 0.945; 95% CI 0.920, 0.971; *P* < 0.001), body weight (OR 0.987; 95% CI 0.979, 0.995; *P* 0.001), and body surface area (OR 0.582; 95% CI 0.369, 0.920; *P* 0.020) were inversely associated with moderate-to-severe coronary calcification (CAC score ≥ 100). This study extends the concept of obesity paradox to symptomatic patients undergoing coronary artery calcium score assessment. However, biological explanation(s) of this paradox remains unanswered.

## 1. Introduction

Obesity has been believed to be one of the major risk factors and adverse prognosticators, associated with increased mortality risk, for atherosclerotic diseases, especially coronary artery disease (CAD) [1, 2]. Extensive studies have shown that obesity is an independent predictor for CAD and cardiovascular death in multiple populations including a large-scale epidemiological study and a systemic review [3, 4]. Obesity is not only associated with prevalence and death in CAD itself but also related to its major risk factors including hypertension, diabetes mellitus, and dyslipidemia [5]. Body mass index (BMI) which is the index most commonly used in majority of the studies to define obesity has also been shown to be positively associated with increased risk of CAD even in the normal weight range [6]. Pathophysiology of obesity and cardiovascular diseases is complicated and it involves several pathways particularly cardiovascular hemodynamics, systemic inflammation, and leptin metabolism [5]. Given its complexity of interaction and arguable robustness of BMI in defining obesity, it is not surprising that evidences of ability of obesity to be a risk factor and a poor prognosticator are inconsistent among studies. There have been multiple studies describing “obesity paradox,” a protective effect of obesity, with various clinical surrogates and outcomes in different populations of CAD including asymptomatic population [5, 7], myocardial infarction [8–10], and patients treated with revascularization [11, 12]. However, in patients with acute chest pain undergoing coronary artery calcification (CAC) evaluation, there have been sparse studies.

This study seeks to primarily examine relationship between obesity and significant CAD in patients with acute chest pain of unknown cardiac significance who were admitted in an observation unit to further support or reject an idea of obesity paradox in this particular population.

## 2. Materials and Methods

### 2.1. Study Population

This study is a prospective observational cohort study conducted from September 2005 to February 2008. Subjects were patients older than 18 years old who were admitted under observational status for further evaluation of acute chest pain suggestive of myocardial ischemia within the previous 24 hours. The evaluation was performed with single-photon emission computed tomography (SPECT) and CAC scoring by multidetector cardiac CT. Exclusion criteria were subjects with noncardiac chest pain based on clinical assessment, elevated troponin on initial blood samples, new or presumably new ST-segment elevation or depression (≥1 mm) on baseline electrocardiogram, hemodynamic or clinical instability defined by systolic blood pressure <90 mmHg or clinical significant atrial/ventricular arrhythmia, history of coronary artery disease based on previous coronary angiography or prior coronary revascularization, and subjects with known or suspected pregnancy. More detailed materials and methods were described elsewhere [13].

### 2.2. Body Morphology and Obesity

Indices for body morphology used in this study were height in meter, weight in kilogram, body surface area (BSA) in meter^2^ calculated by DuBois and DuBois formula [14], and BMI. BMI of each subject was calculated by the following formula: weight in kilogram ÷ (height in meter)^2^. Definition of obesity was set at BMI at least 30 kg/m^2^.

### 2.3. CAC Scoring

CAC score was calculated with reconstructed axial images of 2.5 mm thickness as previously described by Agatston et al. [15]. A 16-slice multidetector CT scanner (Philips Precedence, Philips Healthcare, Eindhoven, The Netherlands) was used. Images were acquired during a single breath hold, using prospective ECG gating with imaging triggered at 75% of the R-R interval. Patients were categorized into 2 groups based on their CAC extent: absent-to-mild CAC (CAC score < 100) or moderate-to-severe CAC (CAC score ≥ 100).

### 2.4. Data Gathering and Processing

During observation period, all clinical information was collected including demographic information, cardiovascular history (i.e., cardiovascular symptoms, history of hypertension, history of diabetes mellitus, history of dyslipidemia, smoking history, history of peripheral arterial disease, history of carotid artery disease, history of abdominal aortic aneurysm, family history of coronary artery disease, and current cardiovascular medication profiles), and blood samples for lipid profiles, cardiac biomarkers, and renal function tests. Clinical information was used to calculate Framingham coronary heart disease 10-year risk score [16], TIMI risk score for acute coronary syndrome [17], and ATP III risk score for coronary artery disease [18].

### 2.5. Statistical Analysis

Descriptive statistics for studied variables are presented as mean with standard deviation (SD) for normally distributed variables, including age, height, weight, BSA, and BMI, median with range for nonnormally distributed variables, including Framingham coronary heart disease 10-year risk score and TIMI risk score for acute coronary syndrome, and frequency with percentage for categorical variables including binary cardiovascular risk factors and uses of cardiovascular medications. Student's *t*-test was used to identify differences in mean. Mann-Whitney *U* test was used to examine differences in medians. *χ* ^2^ analysis was used to identify significant heterogeneity in the frequencies. Binary logistic regression was performed to examine relationship between covariables and presence of moderate-to-severe CAC. Odd ratio and 95% confidence interval (CI) were used to describe the relationship. Body morphology indices that had statistical significant difference between CAC groups were included as testing variables. All baseline characteristics that are significant univariable predictors at the level of *P* < 0.1 were entered into multivariable models for each testing variable to identify ability to be an independent predictor. Framingham CAD risk score was entered into multivariable model to represent age, gender, smoking history, dyslipidemia, and hypertension. Other baseline characteristics that were included are history of stroke and history of PAD. All statistical analyses were performed with IBM SPSS/PASW Statistics 20 (SPSS Inc., Chicago, IL). *P* value < 0.05 was considered statistically significant.

## 3. Results

### 3.1. Study Population Characteristics

A total of 5066 patients presented to our ED with acute chest pain between September 2005 and February 2008. The final cohort consisted of 1030 patients. Studied clinical characteristics are shown in Table 1.

The median CAC score in our study population was 0 with 25th and 75th percentile of 0 and 35.75, respectively. Most (624 of 1030; 60.6%) had absent CAC, followed by 21.7% (223 of 1030) with mild CAC (0 < CAC score < 100) and 17.8% (183 of 1030) with moderate-to-severe CAC (CAC score ≥ 100). Patients with moderate-to-severe CAC had expected results for most clinical characteristics. They were older (*P* < 0.001) and tended to be male (*P* 0.015). They had significantly more history of hypertension (*P* < 0.001), history of dyslipidemia (*P* < 0.001), history of stroke (*P* < 0.001), and history of peripheral arterial disease (PAD) (*P* < 0.001). Other risk factors for CAD were comparable between 2 groups which are history of diabetes (*P* 0.359), history of cigarette smoking (*P* 0.099), and family history of CAD (*P* 0.405). Compared to patients with absent-to-mild coronary calcification, patients with moderate-to-severe coronary calcification had higher median Framingham CAD 10-year risk score (*P* < 0.001), higher median TIMI risk score (*P* < 0.001), higher proportion of high TIMI risk score (*P* < 0.001), and higher proportion of moderately high and high risk in ATP III criteria (*P* < 0.001). There were also significantly higher prevalence of uses of cardiovascular medications including aspirin (*P* < 0.001), angiotensin converting enzyme inhibitors (ACEIs)/angiotensin receptor blockers (ARBs) (*P* < 0.001), beta blockers (*P* < 0.001), calcium channel blockers (*P* < 0.001), and statins (*P* < 0.001) but not diuretics (*P* 0.101) in moderate-to-severe CAC group compared to absent-to-mild CAC group.

### 3.2. Body Morphology Indices and Obesity

Overall, median BMI was 29.2 kg/m^2^ with 25th and 75th percentile of 25.5 kg/m^2^ and 34.2 kg/m^2^, respectively. There were 223 (21.7%) normal weight patients, 336 (32.6%) overweight patients, and 471 (45.7%) obese patients. Other body morphology indices, differences in median BMI, and differences in mean height, mean weight, and mean BSA are shown in Table 2. Patients with moderate-to-severe CAC had significantly lower median body weight (*P* 0.005), BSA (*P* 0.037), and BMI (*P* 0.001) than those with absent-to-mild CAC. Consistently with BMI, percentage of obesity in the moderate-to-severe CAC group was significantly lower than that in absent-to-mild CAC group (*P* 0.010).

Binary logistic regression analysis was used to examine these associations. Results are shown in Table 3. Body weight, BSA, BMI values, and presence of obesity are inverse univariable predictors for moderate-to-severe CAC compared to absent-to-mild CAC (body weight: OR 0.989, 95% CI 0.982, 0.997, *P* 0.005; BSA: OR 0.636, 95% CI 0.415, 0.974, *P* 0.038; BMI: OR 0.956, 95% CI 0.933, 0.981, *P* 0.001; presence of obesity: OR 0.651, 95% CI 0.469, 0.905, *P* 0.011). Multivariable analysis was then evaluated by entering Framingham CAD risk score, history of stroke, and history of PAD into the model to explore confounding effect on each testing univariable predictor. All the testing univariable predictors did not only remain independent predictors for moderate-to-severe CAC compared to absent-to-mild CAC but also showed stronger relationship with greater magnitude in OR and 95% CI (body weight: OR 0.98, 95% CI 0.979,0.995, *P* 0.001; BSA: OR 0.582, 95% CI 0.369,0.920, *P* 0.020; BMI: OR 0.945, 95% CI 0.920,0.971, *P* < 0.001; presence of obesity: OR 0.564, 95% CI 0.395,0.806, *P* 0.002).

## 4. Discussion

Our final cohorts of 1030 patients under observation status for acute chest pain evaluation confirmed the concept of obesity paradox in this population. Other body morphology indices including body weight and BSA also showed inverse relationship with moderate-to-severe CAC as well consistently with a recent coronary angiography-based study. Despite the fact that patients with moderate-to-severe CAC were older and had more history of hypertension, dyslipidemia, stroke, and PAD, body morphology indices including BMI were lower in this group. With adjusted logistic regression analysis, presence of obesity, BMI, body weight, and BSA remained independent predictors for moderate-to-severe CAC. In our study, we did not find significant differences in any body composition indices between patients with and without short-term cardiac events.

We chose to use Framingham CAD risk score for an adjustment as a surrogate for age, gender, history of hypertension, and history of dyslipidemia because, with a well-validated risk score as the Framingham CAD risk score, we aimed to minimize confounding effect and interaction between each other in these factors. We also added history of stroke and history of PAD to the adjusted analysis as they are not included in the Framingham risk score and the differences in these baseline characteristics were also significant. We specifically chose moderate-to-severe CAC defined by CAC score of at least 100 as our cutoff for significant CAD as natural history of CAC is over time progressing as suggested by a recent study [19], so robustness of mild CAC in older age group in identifying clinical significant CAD might be arguable. CAC score of at least 100 has also been shown to be associated with significantly higher risk of increased cardiac events including CAD death, nonfatal myocardial infarction, and unstable angina pectoris requiring coronary revascularization [20, 21].

In contrast to several prior CT-based studies which demonstrated positive relationship between BMI and CAC score [22–28], our study found that increased BMI was associated with lower CAC. However, most of the previous studies were conducted on asymptomatic subjects without significant CAD who were at low risk of developing CAD. There is one CT-based study [28], to our knowledge, that investigated this association in patients with significant CAD; however this study contained small number of subjects (*N* = 115). These discrepancies in findings have been suggested before by Lee et al. that BMI has a U-shaped relationship with CAC [29]. In their report, it was shown that, in young asymptomatic subjects without CAD, initial BMI on enrollment was positively associated with CAC at final follow-up in a linear fashion but BMI at follow-up on years 5, 10, and 15 became progressively U shape. BMI at the final follow-up at year 20 was inversely associated with CAC at final follow-up. Nevertheless, our findings in the present study are consistent with multiple prior coronary angiography-based studies [5, 7, 9–11, 30] which described a paradoxical relationship between CAC and body morphology, mainly BMI as well.

Our study extends the concept of obesity paradox with significant CT-derived CAC to symptomatic patients. The findings in this study support the fact that there is obesity paradox in various CAD populations including stable CAD [7], patients referred for exercise stress testing [11, 31], CAD with acute coronary syndrome requiring coronary angiography [8–10], and CAD requiring revascularization [11, 12]. Whether this concept is an unanswered phenomenon or just an artifact from heterogeneous composition of BMI is still controversial to date. Most studies that described obesity paradox used BMI as a surrogate for obesity. Given that BMI comprises body fat and fat-free mass, “obesity” defined by BMI can theoretically be actually muscle mass which could well explain why subjects with higher BMI in studies had better clinical outcomes or had lower CAC compared to subjects with lower BMI. In evidence, there have been multiple studies focused on cardiorespiratory fitness as it is one of the indices that possibly differentiate higher BMI secondary to body fat from higher BMI secondary to fat-free mass. It has been shown that, in subjects with higher cardiorespiratory fitness, there is no obesity paradox as opposed to subjects with lower cardiorespiratory fitness who demonstrated the paradox [32–34]. Other proposed biological mechanisms to explain inverse relationship between BMI and CAC in significant CAD involve another puzzle called “vascular calcification paradox” proposed by Kovacic et al. [30] which describes the phenomenon that increased bone mineralization is inversely associated with vascular calcification. The basis of this theory begins with the hypothesis that bigger body size necessitates bigger bony skeletal support. Bigger body size has been shown to be positively associated with higher bone mineralization [35]. In linking all these findings and the theory together, Broussard and Magnus proposed that the obesity paradox might be related to the vascular calcification paradox. This paradox seems to possibly involve Klotho gene which has been shown in mice and human to regulate bone mineralization and vascular calcification [36–38]. The possibility of this theory has also been supported in patients who use bisphosphonate which demonstrated increased bone mass but decreased vascular calcification [39].

In contrast to BMI, BSA has not been studied extensively for associations with CAC or cardiac events. BSA is also a product of weight and height as BMI, although it is a product of multiplication as opposed to division in BMI which might not reflect obesity being as robust as BMI. The results of our study support a recent study by Roy et al. [40] which examined a relationship between BSA and CAC in patients who underwent cardiac CT for coronary artery evaluation. However, the relationship between BSA and CAC score was inverse in our study but positive in their study. They also demonstrated that BSA, but not BMI, is an independent predictor for presence of CAC.

The limitation of this study is the single-center cross-sectional nature of examining association between body morphology and CAC. For all body morphology indices including BMI, we used a single measurement for each patient at the time of presentation to correlate with CAC. All the lack of association between body morphology indices and coronary calcium score in our study cannot reflect changes in these indices in temporal fashion. Also we did not use other body morphology measurements such as waist circumference, hip circumference, or waist-to-hip ratio which might show different results.

Perspectives: in contrast to several previous CT-based studies examining the relationship between BMI and CAC which show positive association in asymptomatic subjects, our study is one of the first large-scale studies which demonstrated that the association is reversed in patients with acute chest pain. However, this reverse relationship between CAC and BMI was described before in symptomatic patients, though with CAC detected by angiography. This raises the possibility of obesity paradox with CAC detected by cardiac CT in this population as well as questions regarding robustness between the two imaging modalities in detecting CAC. Also there could possibly be differences in disease stages or biological mechanisms between asymptomatic subjects and symptomatic patients that could potentially explain this discrepancy. Further investigations are needed to confirm this paradoxical association and to find its biological explanation(s).

## 5. Conclusion

In patients with acute chest pain who were admitted under observation status for CAC evaluation, we demonstrated an inverse association between body weight, BSA, and BMI and significant CT-derived CAC. These findings extend the concept of obesity paradox to this population. However, biological explanation(s) of this paradox remains unanswered. Further studies are needed to examine the mechanism of this concept. 

*Novelty and Significance*

*What Is New?*

- Our study is a large-scaled study on symptomatic patients that demonstrated paradoxical association between cardiac CT-derived coronary artery calcification and obesity as well as other body morphology indices.
- Most of the previous cardiac CT-based studies were conducted on asymptomatic subjects and showed positive association between obesity and coronary artery calcification.

*What Is Relevant?*

Obesity paradox in cardiovascular diseases including hypertension has been repeatedly described. Our study extends this concept to a different population with a different imaging modality.

*Summary*

The present study showed that, in patients with acute chest pain admitted for observation, obesity was an inverse independent predictor for coronary artery calcification detected by cardiac CT.

## Figures and Tables

**Table 1:** Baseline characteristics.

Clinical characteristics	All patients (*N* = 1030)	Absent-to-mild CAC (*N* = 847)	Moderate-to-severe CAC (*N* = 183)	*P* value
Mean age, year (SD)	54.0 (13.5)	51.2 (11.8)	66.8 (13.2)	<0.001
Male, no. (%)	413 (40.1)	522 (61.6)	95 (51.9)	0.015

Risk factors for CAD, no. (%)				
History of hypertension	590 (57.3)	454 (53.6)	136 (74.3)	<0.001
History of diabetes	152 (14.8)	121 (14.3)	31 (16.9)	0.359
History of dyslipidemia	352 (34.2)	261 (30.8)	91 (49.7)	<0.001
History of cigarette smoking	192 (18.6)	150 (17.7)	42 (23.0)	0.099
Family history of CAD	52 (5.0)	45 (5.3)	7 (3.8)	0.405
History of stroke	35 (3.4)	19 (2.2)	16 (8.7)	<0.001
History of PAD	12 (1.2)	5 (0.6)	7 (3.8)	<0.001
Framingham risk score, median (range)	4 (1–30)	2 (1–30)	16 (1–30)	<0.001
TIMI risk score, median (range)	1 (1–4)	1 (1–4)	2 (1–4)	<0.001
1, no. (%)	655 (63.6)	600 (70.8)	55 (30.1)	<0.001
2, no. (%)	258 (25.0)	185 (21.8)	73 (39.9)	
3, no. (%)	106 (10.3)	58 (6.8)	48 (26.2)	
4, no. (%)	11 (1.1)	4 (0.5)	7 (3.8)	

ATP III groups, no. (%)				
Low	366 (35.5)	353 (41.7)	13 (7.1)	<0.001
Moderate	305 (29.6)	258 (30.5)	47 (25.7)	
Moderately high	156 (15.1)	97 (11.5)	59 (32.2)	
High	203 (19.7)	139 (16.4)	64 (35.0)	

Baseline cardiovascular medication use, no. (%)				
Aspirin	190 (18.4)	132 (15.6)	58 (31.7)	<0.001
ACEIs/ARBs	325 (31.6)	249 (29.4)	76 (41.5)	0.001
Beta blockers	164 (15.9)	118 (13.9)	46 (25.1)	<0.001
Diuretics	182 (17.7)	142 (16.8)	40 (21.9)	0.101
Calcium channel blockers	139 (13.5)	97 (11.5)	42 (23.0)	<0.001
Statins	240 (23.3)	167 (19.7)	73 (39.9)	<0.001

**Table 2:** Body morphology indices and obesity categorized by extent of CAC.

Body indices	All patients (*N* = 1030)	Absent-to-mild CAC (*N* = 847)	Moderate-to-severe CAC (*N* = 183)	*P* value
BMI, median (range)	29.2 (14.8–68.6)	29.4 (17.4–68.6)	27.5 (14.8–57.8)	0.001
Obesity, no. (%)	471 (45.7)	403 (47.6)	68 (37.2)	0.010
Body weight (kg) (SD)	86.4 (23.2)	87.4 (23.5)	82.1 (21.5)	0.005
BSA (m^2^) (SD)	2.73 (0.39)	2.74 (0.39)	2.67 (0.38)	0.037
Height (meters) (SD)	1.68 (0.11)	1.68 (0.11)	1.68 (0.11)	0.616

**Table 3:** Association between body morphology indices and moderate-to-severe CAC.

Body indices	Univariable analysis	*P* value	Multivariable analysis*	*P* value
BMI	0.956 (0.933, 0.981)	0.001	0.945 (0.920, 0.971)	<0.001
Obesity	0.651 (0.469, 0.905)	0.011	0.564 (0.395, 0.806)	0.002
Body weight	0.989 (0.982, 0.997)	0.005	0.987 (0.979, 0.995)	0.001
BSA	0.636 (0.415, 0.974)	0.038	0.582 (0.369, 0.920)	0.020

*Adjusted for Framingham CAD risk score, and history of stroke, and history of PAD.

## References

[1] Hubert HB, Feinleib M, McNamara PM, Castelli WP (1983). Obesity as an independent risk factor for cardiovascular disease: a 26-year follow-up of participants in the Framingham Heart Study. *Circulation*.

[2] Whitlock G, Lewington S, Sherliker P (2009). Body-mass index and cause-specific mortality in 900 000 adults: collaborative analyses of 57 prospective studies. *The Lancet*.

[3] Wilson PW, D'Agostino RB, Sullivan L, Parise H, Kannel WB (2002). Overweight and obesity as determinants of cardiovascular risk: the Framingham experience. *Archives of Internal Medicine*.

[4] Bogers RP, Bemelmans WJE, Hoogenveen RT (2007). Association of overweight with increased risk of coronary heart disease partly independent of blood pressure and cholesterol levels: a meta-analysis of 21 cohort studies including more than 300 000 persons. *Archives of Internal Medicine*.

[5] Lavie CJ, Milani RV, Ventura HO (2009). Obesity and cardiovascular disease. risk factor, paradox, and impact of weight loss. *Journal of the American College of Cardiology*.

[6] Willett WC, Dietz WH, Colditz GA (1999). Guidelines for healthy weight. *New England Journal of Medicine*.

[7] Lavie CJ, De Schutter A, Patel D, Artham SM, Milani RV (2011). Body composition and coronary heart disease mortality—an obesity or a lean paradox?. *Mayo Clinic Proceedings*.

[8] Zeller M, Steg PG, Ravisy J (2008). Relation between body mass index, waist circumference, and death after acute myocardial infarction. *Circulation*.

[9] Diercks DB, Roe MT, Mulgund J (2006). The obesity paradox in non-ST-segment elevation acute coronary syndromes: results from the Can Rapid risk stratification of Unstable angina patients Suppress ADverse outcomes with Early implementation of the American College of Cardiology/American Heart Association Guidelines Quality Improvement Initiative. *American Heart Journal*.

[10] Buettner HJ, Mueller C, Gick M (2007). The impact of obesity on mortality in UA/non-ST-segment elevation myocardial infarction. *European Heart Journal*.

[11] Romero-Corral A, Montori VM, Somers VK (2006). Association of bodyweight with total mortality and with cardiovascular events in coronary artery disease: a systematic review of cohort studies. *Lancet*.

[12] Lavie CJ, Milani RV (2003). Obesity and cardiovascular disease: the Hippocrates paradox?. *Journal of the American College of Cardiology*.

[13] Nabi F, Chang SM, Pratt CM (2010). Coronary artery calcium scoring in the emergency department: identifying which patients with chest pain can be safely discharged home. *Annals of Emergency Medicine*.

[14] Du Bois D, Du Bois EF (1989). A formula to estimate the approximate surface area if height and weight be known,1916. *Nutrition*.

[15] Agatston AS, Janowitz WR, Hildner FJ, Zusmer NR, Viamonte M, Detrano R (1990). Quantification of coronary artery calcium using ultrafast computed tomography. *Journal of the American College of Cardiology*.

[16] Wilson PWF, D’Agostino RB, Levy D, Belanger AM, Silbershatz H, Kannel WB (1998). Prediction of coronary heart disease using risk factor categories. *Circulation*.

[17] Antman EM, Cohen M, Bernink PJLM (2000). The TIMI risk score for unstable angina/non-ST elevation MI: a method for prognostication and therapeutic decision making. *Journal of the American Medical Association*.

[18] Grundy SM, Cleeman JI, Merz CN (2004). Implications of recent clinical trials for the National Cholesterol Education Program Adult Treatment Panel III guidelines. *Arteriosclerosis, Thrombosis, and Vascular Biology*.

[19] McEvoy JW, Blaha MJ (2010). Progression of coronary artery calcification: not down-and-out. *Archives of Internal Medicine*.

[20] Petretta M, Daniele S, Acampa W (2012). Prognostic value of coronary artery calcium score and coronary CT angiography in patients with intermediate risk of coronary artery disease. *International Journal of Cardiovascular Imaging*.

[21] Pletcher MJ, Tice JA, Pignone M, Browner WS (2004). Using the coronary artery calcium score to predict coronary heart disease events: a systematic review and meta-analysis. *Archives of Internal Medicine*.

[22] Allison MA, Wright CM (2004). Body morphology differentially predicts coronary calcium. *International Journal of Obesity*.

[23] Hsu CH, Chang SGN, Hwang KC, Chou P (2007). Impact of obesity on coronary artery calcification examined by electron beam computed tomographic scan. *Diabetes, Obesity and Metabolism*.

[24] Kramer CK, Von Mühlen D, Gross JL, Barrett-Connor E (2009). A prospective study of abdominal obesity and coronary artery calcium progression in older adults. *Journal of Clinical Endocrinology and Metabolism*.

[25] Kronmal RA, McClelland RL, Detrano R (2007). Risk factors for the progression of coronary artery calcification in asymptomatic subjects: results from the Multi-Ethnic Study of Atherosclerosis (MESA). *Circulation*.

[26] Lee CD, Jacobs DR, Schreiner PJ, Iribarren C, Hankinson A (2007). Abdominal obesity and coronary artery calcification in young adults: the Coronary Artery Risk Development in Young Adults (CARDIA) Study. *American Journal of Clinical Nutrition*.

[27] See R, Abdullah SM, McGuire DK (2007). The association of differing measures of overweight and obesity with prevalent atherosclerosis. The dallas heart study. *Journal of the American College of Cardiology*.

[28] Brown BG, Morse J, Zhao XQ, Cheung M, Marino E, Albers JJ (2001). Electron-beam tomography coronary calcium scores are superior to framingham risk variables for predicting the measured proximal stenosis burden. *American Journal of Cardiology*.

[29] Lee DH, Steffes MW, Gross M (2010). Differential associations of weight dynamics with coronary artery calcium versus common carotid artery intima-media thickness. *American Journal of Epidemiology*.

[30] Kovacic JC, Lee P, Baber U (2012). Inverse relationship between body mass index and coronary artery calcification in patients with clinically significant coronary lesions. *Atherosclerosis*.

[31] McAuley P, Myers J, Abella J, Froelicher V (2007). Body mass, fitness and survival in veteran patients: another obesity paradox?. *American Journal of Medicine*.

[32] McAuley PA, Artero EG, Sui X (2012). The obesity paradox, cardiorespiratory fitness, and coronary heart disease. *Mayo Clinic Proceedings*.

[33] McAuley PA, Kokkinos PF, Oliveira RB, Emerson BT, Myers JN (2010). Obesity paradox and cardiorespiratory fitness in 12,417 male veterans aged 40 to 70 years. *Mayo Clinic Proceedings*.

[34] Goel K, Thomas RJ, Squires RW (2011). Combined effect of cardiorespiratory fitness and adiposity on mortality in patients with coronary artery disease. *American Heart Journal*.

[35] Broussard DL, Magnus JH (2004). Risk assessment and screening for low bone mineral density in a multi-ethnic population of women and men: does one approach fit all?. *Osteoporosis International*.

[36] Arking DE, Becker DM, Yanek LR (2003). KLOTHO allele status and the risk of early-onset occult coronary artery disease. *American Journal of Human Genetics*.

[37] Kuro-o M, Matsumura Y, Aizawa H (1997). Mutation of the mouse klotho gene leads to a syndrome resembling ageing. *Nature*.

[38] Bucay N, Sarosi I, Dunstan CR (1998). Osteoprotegerin-deficient mice develop early onset osteoporosis and arterial calcification. *Genes and Development*.

[39] Elmariah S, Delaney JAC, O’Brien KD (2010). Bisphosphonate use and prevalence of valvular and vascular calcification in women: MESA (The Multi-Ethnic Study of Atherosclerosis). *Journal of the American College of Cardiology*.

[40] Roy SK, Zeb I, Kadakia J, Li D, Budoff MJ (2012). Body surface area is a predictor of coronary artery calcium, whereas body mass index is not. *Coronary Artery Disease*.

